# Olmesartan-Induced Enteropathy: When the Treatment of One Disease Causes Another

**DOI:** 10.7759/cureus.53556

**Published:** 2024-02-04

**Authors:** Sara Santos, Rita S Costa, Sofia Ferreira, Sérgio Gomes Ferreira, Rita Maciel

**Affiliations:** 1 Internal Medicine, Centro Hospitalar de Entre Douro e Vouga, Santa Maria da Feira, PRT

**Keywords:** hypertension, potential celiac disease, chronic diarrhea, enteropathy, olmesartan

## Abstract

Olmesartan is an angiotensin II receptor antagonist used for the management of hypertension. This drug can lead to an enteropathy that clinically and histologically resembles coeliac disease. Symptoms may appear months or years after the introduction of the drug and usually resolve after discontinuation.

The authors present a case of an 86-year-old woman with hypertension who was treated with olmesartan for 10 years. She presented to the emergency department with diarrhoea after three months of development and weight loss. The aetiological study that was conducted excluded infectious, inflammatory, endocrinological, and neoplastic causes. The pathological anatomy of the duodenal biopsy was suggestive of coeliac disease, but the serology was not compatible. The patient presented complete remission of the condition with the suspension of the drug and subsequent recrudescence when, by self-initiation, she resumed olmesartan.

This case study aims to alert readers of a rare cause of enteropathy with a clinical manifestation that mimics coeliac disease. Olmesartan-induced enteropathy seems to be a diagnosis of exclusion and should be considered in patients chronically medicated with olmesartan.

## Introduction

Olmesartan is a drug of the angiotensin II receptor antagonist (ARA II) class widely used in the pharmacological treatment of hypertension, with few side effects described. However, these side effects include olmesartan-induced enteropathy, a rare clinical entity that is present in <1% of regular drug users [1]. Its description in the literature is very recent, with the first series of cases presented in 2012 [2].

Recognising this entity is highly challenging, as it is a diagnosis of exclusion. The most common clinical manifestations are non-bloody chronic diarrhoea, unintentional weight loss, and abdominal discomfort. Histologically, it presents findings consistent with coeliac disease, namely, flattening or atrophy of the duodenal villi, intraepithelial lymphocytosis, and inflammation of the lamina propria [1]. This enteropathy is distinguished from coeliac disease by the absence of compatible serologies and the lack of response to a gluten-free diet. The suspension of ARA II drugs results in a significant and rapid improvement in the symptoms of diarrhoea and progressive resolution of histological findings [2].

The physiopathology of the disease is not yet fully understood, but given the possibility that there is a long period between the onset of exposure and symptoms, it appears that cell-mediated immunity may play a role in this clinical entity [3]. In addition, there appears to be a positive regulation of pro-apoptosis proteins (e.g., Bax and GATA-6) and a negative BCL-2 regulation, leading to apoptosis of intestinal epithelial cells with consequent villous atrophy [3].

## Case presentation

An 86-year-old woman with a history of essential hypertension grade I and hypertensive heart disease, treated with olmesartan at 20 mg/day for 10 years, appeared in the emergency department with three months of development of nausea, diarrhoea (around 15 stools per day, without blood or mucus), and weight loss of 10%. She denied having a fever at home. She had no travel history, recent use of antibiotics, or introduction of new drugs.

At the objective examination, she was dehydrated, hypotensive, tachycardic, and apyretic, with discomfort at palpation of the lower quadrants of the abdomen.

Initial laboratory testing showed acute kidney injury (stage 3 Acute Kidney Injury Network (AKIN)/Kidney Disease: Improving Global Outcomes (KDIGO)), with non-anion gap metabolic acidosis (pH = 7.28, partial pressure of carbon dioxide (pCO2) = 31 mmHg, bicarbonate (HCO3−) = 18.4 mmol/L), severe hypokalaemia (K+ = 2.3 mmol/L), and a discreet increase in inflammatory parameters (C-reactive protein = 45 mg/dL and leukocytosis = 11.5x10^9/L). There was no evidence of anaemia or peripheral eosinophilia, with normal thyroid function.

**Figure 1:**
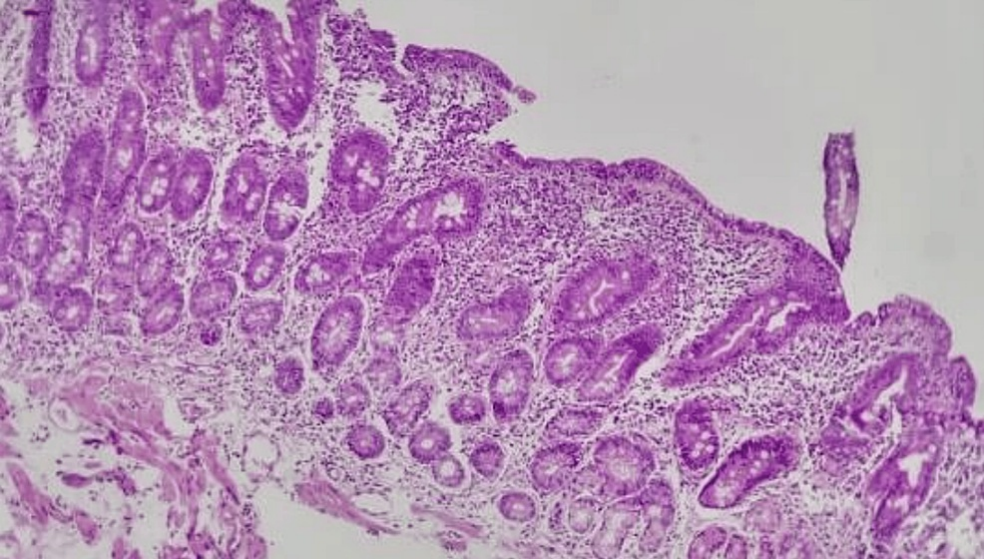
Biopsy of duodenal mucosa demonstrating atrophy of villi and hyperplasia of crypts.

The patient was admitted to the medical intermediate care unit under vigorous fluid therapy and intravenous potassium replacement, with gradual overall improvement. The aetiological study conducted highlighted discreetly elevated faecal calprotectin (587 mcg/g). Parasitological and bacteriological studies of faeces were negative, as well as tests for leukocytes in the faeces and for *Clostridioides* toxin. Serum proteins, albumin, iron kinetics, and dosing of vitamin B12 and folic acid were normal. The serologies for human immunodeficiency virus and hepatitis B and hepatitis C viruses were negative. Tests for faecal elastase, fat, and muscle fibres were negative, as were tests for antibodies of coeliac disease (anti-transglutaminase IgA, anti-endomysial, and anti-gliadin). Abdominal and pelvic computed tomography and upper digestive endoscopy showed no changes with significant pathological interpretation and lower digestive endoscopy showed villous atrophy. An intestinal mucosa biopsy showed histological changes compatible with coeliac disease, namely, an increase in intraepithelial lymphocytes, intestinal crypt hyperplasia, and villous atrophy (Figure 1).

In the hospital, the patient had a favourable clinical and analytical development, with no recrudescence of the diarrhoea. Due to a strong suspicion of enteropathy due to olmesartan, and given that the patient had a controlled hypertension profile in the hospital without any targeted therapy, the patient was discharged to her home without targeted antihypertensive therapy. Having resumed olmesartan at her own initiative at home, the patient again showed a hypertensive profile, with recrudescence of the symptoms and the need for re-hospitalisation three weeks after clinical discharge. On the departure date of the second admission, she was medicated with amlodipine.

## Discussion

Chronic diarrhoea constitutes a diagnostic challenge given the enormous variety and complexity of possible aetiologies for this framework.

Among the differential diagnoses for chronic diarrhoea, it is important to distinguish between osmotic diarrhoea, secretory diarrhoea, malabsorption syndromes, inflammatory diseases of the intestine, ischemic colitis, neoplasms, infectious diarrhoea, and medicated diarrhoea. In this latter entity, it is highlighted that olmesartan is one of the drugs that can lead to chronic diarrhoea; other drugs of less common use and with less impact on this nosological entity include azathioprine, mycophenolate, methotrexate, neomycin, and colchicine [4].

In an extended 2013 study by DeGaetani et al. that included 72 patients with villous atrophy and serology for negative coeliac disease, 26% had drug-induced enteropathy, 16 of which were olmesartan-related [5]. Enteropathy induced by other ARA II drugs is extremely rare, with few cases described related to valsartan, irbesartan, and telmisartan [6].

The data available in the literature do not indicate a gender predominance and show that the age group most affected by olmesartan-induced enteropathy is above the sixth decade of life, as represented by our clinical case [2].

The diagnosis of olmesartan-induced enteropathy is extremely challenging, as there may be a large time gap between the start of the drug and the onset of symptoms, and because it closely resembles coeliac disease from both a clinical and histological point of view. The difference between these entities lies in the absence of serological markers and the response to the suspension of the drug rather than a gluten-free diet.

The pathogenesis of this enteropathy, although not yet fully clarified, includes an immune-mediated inflammatory response with intraepithelial lymphocytosis, thickening of subepithelial collagen, and inflammation of the lining itself [2]. The Mayo Clinic study, led by Rubio-Tapia, further identified a higher prevalence of human leukocyte antigen (HLA)-DQ2 in patients diagnosed with this condition, suggesting that this is a predisposing factor for drug-induced intestinal damage [2].

Treatment of this entity goes through the suspension of the drug and, in severe cases, there may be a place for corticosteroid therapy to relieve the symptoms. Recovery of the duodenal mucosa occurs within a short period of time (around six to eight months) after suspension of olmesartan [2].

## Conclusions

Olmesartan-induced enteropathy, although rare, appears to be increasingly common, given the widespread use of the drug in the treatment of hypertension. Knowledge of this entity is essential to minimise the number of patients who may develop this enteropathy and to avoid more extensive, invasive, and expensive testing of patients hospitalised for chronic diarrhoea. In the present clinical case, the clinical resolution with the suspension of the drug during hospitalisation and the recrudescence of the symptoms with the reintroduction of the medicine at home confirmed the diagnosis.
